# Artificial Intelligence in Cancer Research: Modality Dependence and Limited Visual–Spatial Integration in Multimodal Large Language Models for Breast Cancer Histopathology

**DOI:** 10.3390/life16050763

**Published:** 2026-05-02

**Authors:** Ibrahim Güler, Armin Kraus, Gerrit Grieb, Tevfik Satir, Henrik Stelling

**Affiliations:** 1 Department of Plastic, Aesthetic and Hand Surgery, Otto-von-Guericke University, 39120 Magdeburg, Germany; armin.kraus@med.ovgu.de; 2 Department of Health Management, Friedrich-Alexander-Universität Erlangen-Nürnberg, 90403 Nuremberg, Germany; henrikstelling@googlemail.com; 3 Department of Plastic Surgery and Hand Surgery, Gemeinschaftskrankenhaus Havelhoehe, 14089 Berlin, Germany; gerritgrieb@gmx.de; 4 Department of Plastic Surgery and Hand Surgery, Medical Faculty, RWTH Aachen University, 52074 Aachen, Germany; 5 Center for Dermatosurgery, St. Josefskrankenhaus Heidelberg, Academic Teaching Hospital of the Medical Faculty Mannheim, Heidelberg University, 69115 Heidelberg, Germany; drtevfik@gmail.com; 6 Practices for Nuclear Medicine, 12157 Berlin, Germany

**Keywords:** multimodal large language models, breast cancer histopathology, artificial intelligence, cancer diagnostics, AI safety in oncology, H&E images, segmentation masks, texture dependence, model variability, reproducibility

## Abstract

Multimodal large language models (MLLMs) are increasingly considered for cancer diagnostic support, yet their suitability for histopathological image interpretation remains inadequately characterized. We evaluated six contemporary general-purpose MLLMs (Claude Opus 4.6, Claude Sonnet 4.6, Claude Haiku 4.5, ChatGPT 5.3, Grok 4.2, Gemini 3.1 Pro) on 58 paired hematoxylin and eosin (H&E)-stained breast cancer histopathology images (26 malignant, 32 benign) and corresponding nuclei segmentation masks. Each case was classified five times per model under three conditions, image only (IMAGE), mask only (MASK), and both combined (BOTH), yielding 5220 observations. Mean accuracy dropped from 69.4% (IMAGE) to 49.6% (MASK), below the majority-class baseline of 55.2%. Providing the mask together with the image did not improve classification (68.0%), and for ChatGPT 5.3 produced a net loss of 31 correct predictions. Models maintained elevated mean confidence (67.6) under MASK despite near-random accuracy, and reasoning categories shifted in 67.5% of matched case–run pairs between modalities. Under the conditions tested, current general-purpose MLLMs exhibit strong dependence on visual surface features, fail to effectively integrate spatial structural information, and maintain confidence independent of accuracy. These behavioral limitations are directly relevant to the safe deployment of MLLMs in cancer diagnostic workflows.

## 1. Introduction

Cancer remains one of the leading causes of mortality worldwide, with breast cancer being the most commonly diagnosed cancer in women and a major contributor to cancer-related deaths globally [1]. In histopathology, the evaluation of hematoxylin and eosin (H&E)-stained tissue specimens constitutes a central component of cancer diagnosis, based on the assessment of morphological features by pathologists [2]. The increasing digitization of pathology workflows has enabled the application of computational approaches to histopathological image analysis, with deep learning methods demonstrating promising results in automated classification and segmentation tasks on routinely acquired histological images [2,3,4].

With the advent of multimodal large language models (MLLMs), AI systems capable of integrating diverse multimodal inputs such as text, images, audio, video, and structured clinical data, there has been growing interest in their potential for medical image interpretation beyond the specialized convolutional neural networks (CNNs) that have traditionally dominated this field. Recent studies have shown that MLLMs achieve limited performance in zero-shot classification of cancer histopathology images, but can reach competitive accuracy when augmented with in-context learning [5]. Multi-model comparative evaluations in radiology have revealed substantial inter-model variability in diagnostic accuracy [6,7,8], while direct comparisons indicate that MLLMs remain limited in image-based diagnostic performance; for example, GPT-4V and Gemini Pro Vision reached only 41–49% and 29–39% overall accuracy, respectively, which is below the performance of eight subspecialty-trained radiologists [8]. Beyond accuracy, the reliability of model-generated confidence estimates has emerged as a broader concern in clinical AI. In large language models (LLMs), verbalized confidence has been shown to systematically overestimate actual model accuracy [9], and medical hallucinations, defined as plausible but incorrect model outputs, have been increasingly recognized as a potential patient safety risk in clinical AI applications [10].

Despite these advances, a fundamental question remains largely unexplored: what visual information do MLLMs actually use when classifying histopathological images? For CNNs, Geirhos et al. demonstrated that ImageNet-trained architectures rely predominantly on texture rather than shape information, a finding with substantial implications for model robustness and generalization [11]. Whether MLLMs, which differ fundamentally from CNNs in architecture, training paradigm, and intended use, exhibit analogous behavior remains insufficiently characterized in histopathology. The present study addresses this question through modality manipulation, comparing classification behavior under full visual input (H&E images) and reduced structural input (segmentation masks).

This question is clinically relevant, as histopathological classification relies on the integration of multiple information types, including texture-related cues such as staining patterns and chromatin appearance, color-based features from H&E staining, and structural information such as nuclear morphology, glandular organization, and spatial cell distribution [2,4,12]. A systematic imbalance in how these information types are utilized could represent a limitation with implications for the contexts in which such models can be meaningfully deployed.

To address this gap, we designed a controlled feature-ablation framework using paired image–mask data from the UCSB Bio-Segmentation Benchmark, a dataset originally developed for nuclei segmentation evaluation that provides matched histopathology images and corresponding binary segmentation masks for the same cases. First released in 2008, and remaining in active use through systematic reviews and new methodological work into 2025–2026, the dataset has accumulated nearly two decades of published classification and segmentation baselines against which new approaches can be directly compared. Beyond its original use for segmentation benchmarking, prior studies have also employed this dataset for nuclei segmentation, binary malignant-versus-benign classification with handcrafted, hybrid, and deep learning-based feature pipelines, and computer-aided diagnosis frameworks, with reported binary classification accuracies reaching up to 96.7% and area under the curve (AUC) values up to 0.983 for hybrid handcrafted-and-CNN feature fusion approaches [12,13,14,15,16,17,18,19,20,21,22,23,24,25,26,27,28,29].

Critically for the present study, the UCSB benchmark is among the few extensively studied histopathology datasets with an established body of prior classification and segmentation baselines that also provides both H&E images and corresponding segmentation masks for the identical cases, enabling a controlled ablation of visual and spatial information without confounds from dataset-level differences in staining, acquisition, or case selection. Using this setup, we systematically assessed how visual and spatial information contribute to MLLM classification behavior.

## 2. Materials and Methods

### 2.1. Study Design

This cross-sectional exploratory benchmarking study evaluated the modality-dependent classification behavior of six MLLMs across three input conditions. Each of 58 histopathology cases was presented to each model five times per condition, yielding 5220 total observations. The study aimed to characterize how input modality affects diagnostic behavior rather than to establish clinical validation. The experimental design implements a controlled feature-ablation framework at the case level, allowing isolation of visual versus spatial information processing within identical samples.

### 2.2. Dataset

The dataset was drawn from the UCSB Bio-Segmentation Benchmark [13,14], publicly available at the Center for Bio-Image Informatics, UC Santa Barbara [18]. Although primarily introduced as a segmentation benchmark, the dataset has been extensively used for breast cancer classification tasks in prior work [13,14,15,16,17,18,19,20,21,22,23,24,25,26,27,28,29]. Critically, the dataset provides paired image–mask data for each case, a structure that standard histopathology classification datasets do not offer and that is required to manipulate input modality while holding case identity constant. The complete dataset comprised 58 H&E-stained histopathology cases of breast tissue, classified as malignant (*n* = 26, 44.8%) or benign (*n* = 32, 55.2%) yielding a majority-class baseline accuracy of 55.2%. Ground truth labels were provided with the original dataset. Accompanying segmentation masks represented partial annotations of cell nuclei that do not capture all nuclei within each image, rather than exhaustive expert delineations. The masks contain no information about glands, stroma, or tissue context. As the dataset is publicly available, including through third-party mirrors on data-science platforms, prior exposure during model training cannot be excluded [28,29].

### 2.3. Experimental Conditions

Three experimental conditions were defined to isolate the effect of input modality on classification behavior (Table 1). In the IMAGE condition, models received only the H&E-stained histopathology image, providing access to tissue architecture, nuclear morphology, cell distribution, and staining patterns. In the MASK condition, models received only the binary segmentation mask, limiting available information to spatial distribution of annotated regions without color, texture, or nuclear detail. In the BOTH condition, models received the histopathology image together with its corresponding segmentation mask overlay.

### 2.4. Models

Six MLLMs were evaluated: Claude Opus 4.6, Claude Sonnet 4.6, and Claude Haiku 4.5 (Anthropic, San Francisco, CA, USA); ChatGPT 5.3 (OpenAI, San Francisco, CA, USA); Grok 4.2 (xAI, San Francisco, CA, USA); and Gemini 3.1 Pro (Google DeepMind, Mountain View, CA, USA). All models were accessed via their respective web interfaces using default settings without modification of API parameters. Version status reflects March 2026. The selection of multiple contemporary MLLMs follows the multi-model comparative approach established in prior diagnostic evaluation studies [6,7,8].

### 2.5. Prompting Strategy

An identical zero-shot prompt was used for each condition and presented to all models. Models were instructed to produce a forced output consisting of three components: a binary classification label (malignant or benign), a confidence score on a 0–100 scale, and a reasoning category selected from five predefined options: Nuclear features, Global structure, Texture, Cell distribution, or Uncertain guess. Global structure was defined as the tissue-level architectural pattern, including glandular organization, stromal arrangement, and overall tissue morphology. The five reasoning categories were defined a priori to cover established histopathological feature domains, with an ‘Uncertain guess’ option to avoid forcing confident attributions in ambiguous cases. A constrained forced-choice format was deliberately chosen over free-text outputs: forced-choice responses are directly parsable and statistically comparable across models and conditions, while free-text analysis would additionally require subjective post hoc coding. Given that confabulation and hallucination are well-documented inherent limitations of MLLMs in medical contexts [10], restricting the output space to predefined categories was adopted as a conservative design choice to reduce the surface area on which such behavior could manifest in the reasoning outputs. Reasoning categories reflect constrained categorical output selections and are treated as behavioral outputs describing which feature domain the model nominally invoked, not as explanations of internal model cognition or decision processes. Following Albaqshi et al. [7], each case was presented five times per model and condition to assess intra-model consistency. Procedural details on session handling and inference are described in the following paragraph.

Images and masks were obtained from the UCSB Bio-Segmentation Benchmark and converted from their original TIFF distribution format to PNG by lossless format conversion, required to enable upload via the web interfaces of the evaluated models; as both TIFF and PNG are lossless formats, this conversion preserves all pixel values without resampling, resizing, or color modification. No further preprocessing was applied [13,14,18]. For each case and condition, the corresponding file was attached via the standard image-attachment function of the respective web interface in a single user turn together with the textual prompt. Each of the five repeated runs per model–condition cell was conducted in a separate browser session without conversational memory or cached context, and no prior history was available to the model between runs. All inferences were performed in March 2026. All 5220 model outputs complied with the predefined forced-choice output format; no non-compliant or refused responses were observed across the experiment. The full prompt is provided as Supplementary Material S1.

### 2.6. Outcome Definitions

The primary outcome was binary classification accuracy (malignant vs. benign). Secondary outcomes included inter-run reliability, confidence calibration, reasoning category distributions, and pairwise label changes between the IMAGE and BOTH conditions. Two levels of aggregation were applied throughout the analysis. Run-level analysis (*n* = 290 per model–condition cell) treated each of the five repeated observations per case as an independent data point. Run-level results are interpreted descriptively and were used for accuracy estimation, confidence interval (CI) computation, confidence calibration, and reasoning category analysis. Run-level aggregation was retained to increase measurement stability, while inferential comparisons were restricted to case-level (majority-vote) analysis to ensure independence. Majority-vote analysis (*n* = 58) assigned each case the most frequently predicted label across five runs and was used for computing sensitivity, specificity, positive predictive value (PPV), negative predictive value (NPV), balanced accuracy, and McNemar’s test comparisons.

### 2.7. Statistical Analysis

Classification accuracy was calculated as the proportion of correct predictions at the run level. Wilson score intervals [30] were used for 95% confidence intervals on run-level proportions. Because repeated runs within the same case are not statistically independent, run-level confidence intervals and standard deviations are interpreted descriptively and reflect sampling variability rather than cluster-corrected population estimates. Building on the paired-comparison framework used in prior MLLM benchmarking studies (e.g., Sonoda et al. [6]), McNemar’s exact test [31] (two-sided binomial) was applied to majority-vote classifications (*n* = 58) for two within-model comparisons per model (IMAGE vs. MASK and IMAGE vs. BOTH), yielding 12 tests. Bonferroni correction was applied with a familywise threshold of α = 0.05/12 = 0.0042; uncorrected *p*-values are reported throughout, with the corrected threshold noted.

Fleiss’ kappa (κ) [32] was computed for each model–condition combination using 58 cases rated by five repeated runs, treating each run as an independent rater [7]. As repeated model inferences do not constitute independent raters in the psychometric sense, κ values are reported descriptively as a measure of intra-model consistency rather than formal inter-rater agreement. Kappa values were interpreted according to the benchmarks of Landis and Koch [33]:

- Poor (κ < 0.00);
- Slight (0.00–0.20);
- Fair (0.21–0.40);
- Moderate (0.41–0.60);
- Substantial (0.61–0.80);
- Almost perfect (0.81–1.00).

Label flip rate was defined as the proportion of cases with at least one divergent label across five runs.

Confidence calibration was assessed descriptively by comparing mean confidence scores for correct versus incorrect predictions, with standard deviations (SD) computed across all individual observations. Formal calibration metrics (e.g., expected calibration error) were not applied given the exploratory nature of the study and the known limitations of verbalized confidence as an uncertainty proxy, which has been shown to be systematically overconfident [9]. Reasoning shift rate was defined as the proportion of matched case–run pairs with different reasoning categories between IMAGE and MASK conditions. Pairwise label change analysis compared matched IMAGE–BOTH observation pairs, classifying each as improved (incorrect → correct), worsened (correct → incorrect), or unchanged. Diagnostic performance metrics (sensitivity, specificity, PPV, NPV, balanced accuracy) were computed on majority-vote predictions (*n* = 58). All analyses were conducted in Python 3.12 using standard visualization and statistical libraries.

## 3. Results

### 3.1. Classification Accuracy

**Figure 1:**
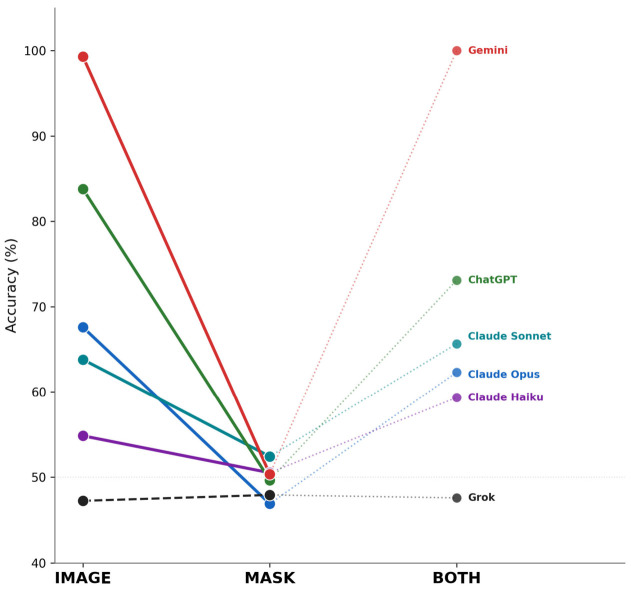
Classification accuracy by model and experimental condition. Slope chart showing run-level accuracy (%) for each of six MLLMs across IMAGE, MASK, and BOTH conditions (*n* = 290 per model–condition cell). Solid lines connect IMAGE to MASK; dotted lines connect MASK to BOTH. All models with above-chance IMAGE performance converged toward near-random accuracy (~50%) under the MASK condition. Grok 4.2 (dashed line) operated near the majority-class baseline across all conditions, consistent with near-constant malignant classifier behavior.

Across all models, mean run-level accuracy was 69.4% (95% CI 67.2–71.5%) for IMAGE, 49.6% (95% CI 47.3–52.0%) for MASK, and 68.0% (95% CI 65.8–70.1%) for BOTH (Table 2, Figure 1). The MASK condition reduced accuracy to near-random performance (~50%), below the majority-class baseline of 55.2%, for all models. The largest absolute accuracy drops from IMAGE to MASK were observed for Gemini 3.1 Pro (−49.0 percentage points, from 99.3% to 50.3%), ChatGPT 5.3 (−34.1 percentage points, from 83.8% to 49.7%), and Claude Opus 4.6 (−20.7 percentage points, from 67.6% to 46.9%). This pattern was observed across all models with above-chance IMAGE performance; models operating near baseline (Grok 4.2) showed minimal change.

After Bonferroni correction (α = 0.0042), the IMAGE-to-MASK accuracy decline reached significance for ChatGPT 5.3 (*p* = 0.0003) and Gemini 3.1 Pro (*p* < 0.0001). Claude Opus 4.6 showed a nominally significant decline (*p* = 0.0081) that did not survive correction. No IMAGE-to-BOTH comparison reached significance for any model. Grok 4.2 operated near baseline across all conditions (47.2–47.9%), consistent with a near-constant malignant classifier (Table 3).

### 3.2. Diagnostic Performance

Table 3 reports sensitivity, specificity, PPV, NPV, and balanced accuracy based on majority-vote predictions (*n* = 58). Under the IMAGE condition, most models exhibited a malignant bias: Claude Sonnet 4.6 and ChatGPT 5.3 achieved perfect sensitivity (100.0%) at the cost of reduced specificity (34.4% and 68.8%, respectively). Claude Haiku 4.5 was the exception, displaying a benign bias with low sensitivity (26.9%) but relatively high specificity (78.1%). Grok 4.2 classified all cases as malignant across IMAGE and BOTH conditions (sensitivity 100.0%, specificity 0.0%), confirming degenerate classifier behavior. Gemini 3.1 Pro achieved perfect diagnostic performance under IMAGE and BOTH (sensitivity 100.0%, specificity 100.0%, balanced accuracy 100.0%) but collapsed to chance under MASK (balanced accuracy 50.0%).

### 3.3. Limited Effective Integration of Combined Input

The BOTH condition did not yield consistent improvement over IMAGE alone (Table 4). Overall BOTH accuracy (68.0%) was marginally lower than IMAGE (69.4%). ChatGPT 5.3 showed a net negative effect, with 42 worsened predictions against 11 improved (net −31). Gemini 3.1 Pro effectively ignored the mask in the combined condition, changing only 2 of 290 matched pairs (0.7%), both of which were improvements. No IMAGE-versus-BOTH McNemar comparison reached significance for any model.

### 3.4. Confidence Calibration

**Figure 2:**
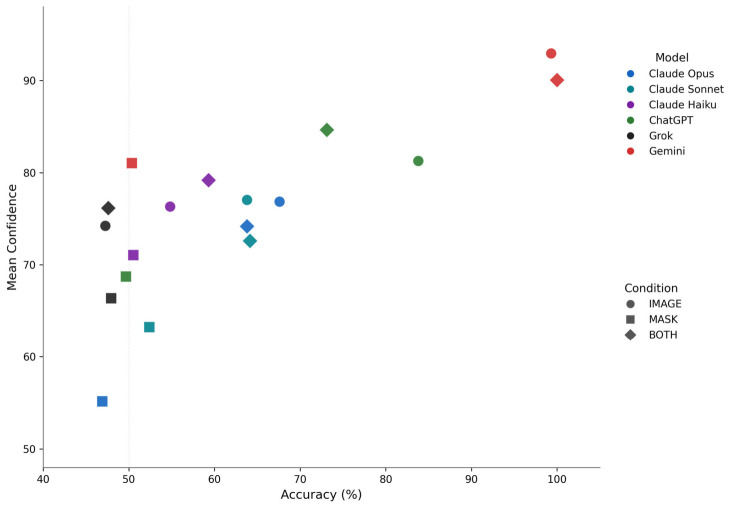
Confidence calibration across experimental conditions. Scatter plot of mean confidence score versus classification accuracy for each model–condition combination. Points are color-coded by model and shape-coded by condition (circle = IMAGE, square = MASK, diamond = BOTH). Under IMAGE and BOTH, data points spanned a wide accuracy range with confidence scores of 73–93. Under MASK, models clustered near 50% accuracy with confidence scores of 55–81, reflecting elevated confidence under near-random performance.

Mean confidence scores were 79.8 (SD = 9.8) for IMAGE, 67.6 (SD = 10.3) for MASK, and 79.5 (SD = 9.1) for BOTH. The difference between mean confidence for correct versus incorrect predictions was +5.6 for IMAGE and +5.0 for BOTH, indicating weak but directionally appropriate calibration. Under MASK, this difference was −0.8, indicating no meaningful calibration: models expressed similar confidence regardless of correctness (Figure 2). MASK-condition data points occupied the left region of the accuracy–confidence space, reflecting elevated confidence across most models under near-random performance, with substantial between-model variability (55–81).

### 3.5. Intra-Model Variability

**Figure 3:**
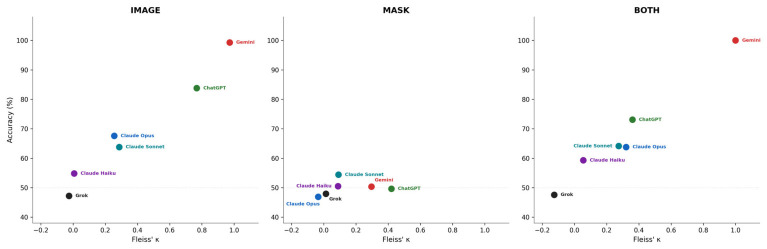
Relationship between classification accuracy and intra-model consistency. Scatter plots showing run-level accuracy (%) versus Fleiss’ κ for each of six MLLMs under IMAGE (**left**), MASK (**center**), and BOTH (**right**) conditions (*n* = 58 cases, five inference runs per case). Each point represents one model. Under IMAGE, models spanned a wide range of both accuracy (47–99%) and consistency (κ = −0.025 to 0.972). Under MASK, all models clustered near 50% accuracy with uniformly low κ values (≤0.420).

Fleiss’ κ ranged from −0.025 to 0.972 for IMAGE, −0.034 to 0.420 for MASK, and −0.125 to 1.000 for BOTH (Figure 3). Under IMAGE, Gemini 3.1 Pro showed almost perfect consistency (κ = 0.972) and ChatGPT 5.3 showed substantial agreement (κ = 0.768), whereas Claude Haiku 4.5 (κ = 0.007, slight) and Grok 4.2 (κ = −0.025, poor) showed near-random variability. Under MASK, all κ values fell below 0.420 (moderate or lower), with the highest consistency observed for ChatGPT 5.3 (κ = 0.420, moderate). Gemini 3.1 Pro exhibited the largest stability loss from IMAGE to MASK (Δκ = −0.676), while Claude Haiku 4.5 remained unstable across all conditions (κ = 0.007–0.089).

Aggregate label flip rates were 40.5% for IMAGE, 76.4% for MASK, and 53.7% for BOTH. At the model level, Gemini 3.1 Pro had the lowest IMAGE flip rate (3.4%, 2/58 cases) and the only zero BOTH flip rate (0.0%), while Claude Haiku 4.5 had the highest IMAGE flip rate (86.2%).

### 3.6. Reasoning Category Analysis

**Figure 4:**
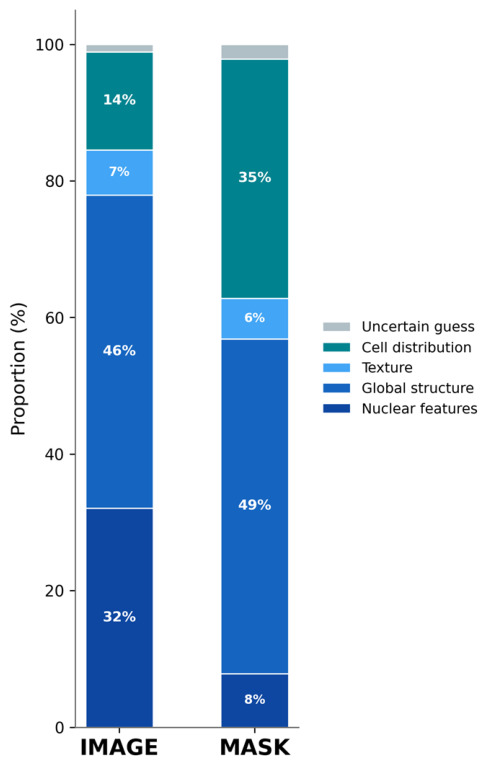
Reasoning category distributions by experimental condition. Stacked bar chart showing the proportion of five predefined reasoning categories (Nuclear features, Global structure, Texture, Cell distribution, Uncertain guess) across all models for IMAGE and MASK conditions. Aggregated across all models (*n* = 1740 observations per condition). Reasoning categories shifted substantially between conditions, with Nuclear features decreasing from 32.1% (IMAGE) to 7.9% (MASK) and Cell distribution increasing from 14.4% (IMAGE) to 35.1% (MASK).

Reasoning category distributions shifted substantially between IMAGE and MASK conditions (Figure 4). Aggregated across all models, Nuclear features accounted for 32.1% of IMAGE responses but only 7.9% of MASK responses. Conversely, Cell distribution increased from 14.4% (IMAGE) to 35.1% (MASK). Global structure remained the most frequently cited category under both conditions (IMAGE: 45.9%; MASK: 49.0%). Texture was rarely cited under either condition (IMAGE: 6.6%; MASK: 6.0%), despite its expected relevance when visual information was removed. Texture dependence is inferred from performance degradation under modality constraints rather than from self-reported reasoning categories. Across all models, 62.1–73.1% of matched case–run pairs exhibited a different reasoning category between IMAGE and MASK (overall: 67.5%).

### 3.7. Error-Type Reasoning

Reasoning categories exhibited class-specific patterns for correct predictions. True positive classifications (correctly identified malignant cases) were dominated by Nuclear features (IMAGE: 52.7% of *n* = 670; BOTH: 61.2% of *n* = 636), while true negative classifications (correctly identified benign cases) were dominated by Global structure (IMAGE: 78.6% of *n* = 538; BOTH: 80.4% of *n* = 547).

False positive predictions (malignant label assigned to benign cases) were predominantly attributed to Nuclear features under IMAGE (43.4% of *n* = 422 false positives) and BOTH (61.5% of *n* = 413). False negative predictions (benign label assigned to malignant cases) were dominated by Global structure under IMAGE (83.6% of *n* = 110) and BOTH (54.2% of *n* = 144). The addition of mask information in the BOTH condition amplified the Nuclear features attribution for false positives (IMAGE: 43.4% → BOTH: 61.5%). Under MASK, error reasoning shifted toward Cell distribution for both false positives (43.5% of *n* = 559) and Global structure for false negatives (61.8% of *n* = 317).

## 4. Discussion

This study employed a controlled feature-ablation framework to characterize the modality-dependent classification behavior of six MLLMs in breast cancer histopathology. The principal findings converge on a central observation: model behavior appears driven by the format and content of the input representation rather than by a stable, modality-independent interpretation of the underlying pathology. Four specific patterns support this conclusion:

- Modality dependence;
- Limited effective integration of spatial structural information;
- Confidence miscalibration under informational constraints;
- Systematic input-dependent reasoning shifts.

### 4.1. Modality Dependence and the Role of Visual Information

The consistent reduction to near-random performance (~50%) under the MASK condition across all models with above-chance IMAGE performance indicates strong dependence on the visual information present in H&E images for classification. This pattern extended across architecturally diverse models from four independent developers, suggesting a shared behavioral characteristic of current MLLMs rather than an idiosyncrasy of any individual system. The magnitude of the performance decline, up to 49 percentage points for Gemini 3.1 Pro, leaves little ambiguity regarding the role of visual surface features in driving classification.

These findings parallel the texture bias documented for CNNs by Geirhos et al. [11]. In that study, a ResNet-50 classified an image with a cat’s shape and elephant-skin texture (generated via style transfer) as ‘Indian elephant’ with 63.9% confidence under texture–shape cue conflict, in stark contrast to human observers across 48,560 psychophysical trials, demonstrating that ImageNet-trained architectures rely predominantly on surface texture rather than object shape. Although the experimental paradigms differ, Geirhos et al. dissociated texture and shape using cue-conflict stimuli, whereas the present study employs modality ablation to dissociate visual from spatial information, the behavioral convergence between their CNN findings and the present MLLM results suggests that visual surface features play a dominant role in image classification across architecturally distinct model classes, extending the texture-bias observation beyond the CNNs originally studied by Geirhos et al. to MLLMs operating under zero-shot conditions.

Classical approaches to classification on the UCSB dataset have explicitly relied on integrated texture, geometric, and color features [17], reaching test accuracies of approximately 90%. Comparative analyses on the same UCSB dataset have shown that the choice of color space (specifically H&E stain decomposition versus RGB, HSV, or L*a*b*) significantly affects breast cancer classification performance, indicating that color information contributes substantial discriminative power when combined with morphological and structural features [12]. The collapse of MLLM performance under removal of visual and color information is consistent with a reliance on similar cues, although, unlike classical pipelines with explicit feature extraction, the specific feature representations leveraged by MLLMs cannot be directly inspected. Notably, despite the apparent role of texture in driving performance, the Texture reasoning category was cited in only 6–7% of responses across conditions, a dissociation between observed performance patterns and self-reported explanations that is itself a key finding and is discussed further in Section 4.4.

### 4.2. Limited Effective Integration of Structural Information

In this study, limited effective visual–spatial integration is defined operationally as the absence of measurable performance improvement when spatial structural information is added to the same visual input. The availability of additional spatial structural information in the BOTH condition did not translate into improved classification performance. Overall BOTH accuracy (68.0%) was marginally lower than IMAGE alone (69.4%), and no IMAGE-versus-BOTH comparison reached significance for any model. For ChatGPT 5.3, the addition of mask information produced a net loss of 31 correct predictions, indicating that the structural overlay actively interfered with classification rather than providing complementary signal.

This finding was unexpected. If models could leverage spatial structural information, the BOTH condition, which provides a strict superset of the information available under IMAGE, should yield at least equivalent and potentially superior performance. Instead, the results suggest that the structural overlay introduced noise or conflicting cues that models were unable to resolve. The pairwise analysis revealed that mask addition amplified Nuclear features attribution in false positive errors (43.4% → 61.5%), consistent with the structural overlay biasing models toward over-detecting nuclear abnormalities.

The contrast with specialized computational approaches is instructive. Dedicated methods that explicitly model structural information through task-specific architectures have achieved strong results on the UCSB dataset, including graph convolutional network-based nuclei segmentation reaching 90.75% accuracy (Dice 83.74%) [16] and deep classification pipelines exceeding 96% accuracy [20,26]. These approaches demonstrate that structural and visual information can be effectively integrated when the architecture is designed for the task. The inability of general-purpose MLLMs to achieve comparable integration under zero-shot conditions highlights a gap between general-purpose multimodal reasoning and domain-specific visual–spatial analysis, and suggests that effective use of spatial structural cues in general-purpose MLLMs may require either task-specific architectural design or substantial adaptation strategies such as in-context learning or fine-tuning, approaches for which preliminary evidence of effectiveness on histopathology classification tasks has been reported [5].

It is important to note that the masks used in this study represent partial annotations rather than exhaustive delineations and contain no information about glands, stroma, or tissue context. Crucially, however, the central finding of this section does not depend on the completeness of the masks. Under the BOTH condition, the visual information available to the models is identical to the IMAGE condition, with the mask provided as an additional source of information on top. Any spatial structural cues present in the mask, whether complete or partial, could therefore only add to the information already available. The absence of any performance gain under BOTH, and the active performance loss observed for ChatGPT 5.3, indicate that the mask information was not effectively integrated, regardless of whether the mask annotations were exhaustive.

### 4.3. Confidence Miscalibration

Under the MASK condition, models expressed mean confidence scores of 67.6 while achieving near-random accuracy (~50%), with virtually no discrimination between correct and incorrect predictions (confidence difference: −0.8). This represents a regime of high confidence under near-random performance, a pattern of confidence miscalibration with particular relevance in medical contexts where model-expressed certainty may influence downstream decision-making.

This finding is consistent with prior work showing that verbalized confidence systematically overestimates LLM accuracy [9]: in that study, sample-consistency-based uncertainty proxies achieved only moderate discriminative performance (ROC AUC 0.68–0.79) across open-ended clinical scenarios, and verbalized confidence elicitation was reported to consistently overestimate model confidence. Our MASK-condition observation, with elevated confidence (mean 67.6) and no discrimination between correct and incorrect predictions (Δ = −0.8), represents a more extreme form of the same miscalibration pattern, occurring at near-random accuracy rather than above-chance performance. This aligns with broader concerns about the reliability of model-generated outputs in clinical AI systems [10].

Recent systematic evaluation of medical hallucinations in foundation models reported a median of 76.6% hallucination-free responses across seven hallucination tasks for general-purpose models, with physician audits attributing 64–72% of residual errors to causal or temporal reasoning failures rather than knowledge gaps [10]. Our observation that reasoning categories shifted in 67.5% of matched case–run pairs while classification accuracy collapsed, in the absence of any underlying change to the diagnostic question, is consistent with this reasoning-failure pattern and suggests that the observed limitation in the present study extends beyond knowledge deficits to instability in reasoning under input manipulation, specifically in the visual–spatial domain.

Importantly, overconfidence has been identified as a broader property of deep learning models trained on small-scale medical imaging datasets [3], suggesting that confidence miscalibration in this domain is not unique to MLLMs but may be amplified by the combination of zero-shot inference and informational constraints.

Under the IMAGE and BOTH conditions, a weak but directionally appropriate calibration was observed (confidence differences of +5.6 and +5.0, respectively), indicating that when sufficient visual information is available, models show some capacity to modulate confidence in relation to accuracy, albeit far from the level required for reliable decision support. Mitigation strategies, including confidence gating, ensemble methods, and uncertainty-aware prompting, were not evaluated in the present zero-shot benchmarking design and represent a relevant direction for future work in this context.

### 4.4. Input-Dependent Reasoning Shifts

Across all models, 67.5% of matched case–run pairs exhibited a different reasoning category when the same case was presented as an image versus a mask. This systematic shift indicates that output explanations are strongly dependent on available input modality rather than reflecting a stable underlying decision strategy. Models adapted their stated reasoning to the information characteristics of the input: Nuclear features dominated under IMAGE but was largely replaced by Cell distribution under MASK, while Global structure remained prevalent across both conditions.

This pattern is consistent with input-dependent rather than stable diagnostic reasoning. The models did not apply a fixed interpretive framework across modalities; instead, they generated condition-appropriate explanations that shifted with the available input. The reasoning categories thus appear to describe the input rather than to explain the decision, a distinction with implications for the interpretability of MLLM-generated justifications in medical contexts. Notably, Texture was cited in only 6–7% of responses across conditions despite the observed performance collapse when visual texture was removed. This dissociation between performance-implied and self-reported feature use mirrors a broader concern that model-generated explanations may function as post hoc rationalizations rather than faithful accounts of the underlying decision process and constrains the use of such explanations as interpretability tools in diagnostic support systems.

The class-specific reasoning structure further supports this interpretation. True positive classifications were consistently driven by Nuclear features (53–61%), while true negative classifications were driven by Global structure (79–80%). This dichotomy, detecting malignancy through nuclear abnormalities and benignity through apparent tissue normality, mirrors pathological reasoning heuristics but was applied inconsistently, as evidenced by the same reasoning categories dominating error patterns: Nuclear features for false positives (models misattributing malignancy to nuclear features in benign cases) and Global structure for false negatives (models falsely reassured by apparent tissue normality).

### 4.5. Model-Specific Observations

The six evaluated models exhibited markedly different behavioral profiles. Gemini 3.1 Pro achieved near-perfect accuracy under IMAGE (99.3%) and BOTH (100.0%) but collapsed to chance under MASK (50.3%), representing the most extreme modality dependence profile. While this high performance is consistent with possible prior training data exposure given the public availability of the UCSB dataset, including through third-party mirrors [28,29], training-data status cannot be directly verified for any of the evaluated models, and any inference about contamination is necessarily indirect. The collapse of performance under MASK argues against a purely memorization-based explanation. Notably, filenames in the UCSB dataset, as well as accompanying XML annotation files distributed alongside the dataset in several repositories, explicitly encode the diagnostic label, and these label-carrying metadata are attached to both the original H&E images and the corresponding segmentation masks. A model whose performance was driven primarily by incidental exposure to these label-tagged files would therefore be expected to classify masks with comparable accuracy to images, since the same filename-based label signal is available in both cases. The observed collapse of Gemini’s performance under MASK (50.3%), together with the absence of any net gain under BOTH despite the mask containing no new visual content beyond the image, is difficult to reconcile with such a memorization account. A more likely interpretation is that training exposure strengthened the visual feature representation specific to this dataset without enabling corresponding spatial reasoning, and that differential exposure to label-tagged metadata across model providers, while not verifiable and not directly claimed, would not by itself explain the observed modality-dependent pattern.

ChatGPT 5.3 demonstrated strong IMAGE performance (83.8%) but was the model most negatively affected by mask addition (net −31 predictions), suggesting that spatial structural information actively interfered with an otherwise effective classification strategy. Grok 4.2 operated as a near-constant malignant classifier across all conditions (47.2–47.9%), producing degenerate output behavior that precluded meaningful interpretation of modality-dependent effects. This model is not considered in model-level interpretations of modality-dependent performance.

### 4.6. Comparison with Prior Literature

Our IMAGE-condition accuracies (47–99% across models) are consistent with prior work demonstrating both limited zero-shot and improved few-shot performance of MLLMs on cancer histopathology classification and the wide inter-model variability mirrors findings from multi-model radiology benchmarks [5,6,8]. Notably, rank ordering across model families is not stable across domains: Claude 3 Opus has been reported to outperform GPT-4o in radiology diagnostic tasks [6], while in our histopathology task ChatGPT 5.3 exceeded all three Claude variants under IMAGE. This cross-task difference suggests that benchmarks on one medical imaging domain cannot be used to predict performance on another, and that model selection for clinical deployment should be based on domain-specific evaluation rather than on general-purpose leaderboards.

The intra-model consistency observed in our study (Fleiss’ κ range: −0.025 to 0.972 under IMAGE) contrasts with the high inter-run stability reported by Albaqshi et al. for neuroradiology cases [7], a discrepancy likely reflecting differences in task format (binary histopathology classification versus multiple-choice neuroradiology questions) and image characteristics. Albaqshi et al. also reported a directly relevant modality-dependence observation: while their best-performing model achieved strong accuracy on combined text-and-image inputs, accuracy for identifying pathologic locations from image-only input dropped substantially across all six tested models [7]. This mirrors the modality-dependence pattern documented in the present study and suggests that the reduction in accuracy when visual or textual context is removed is not confined to histopathology but represents a broader behavioral characteristic of current MLLMs applied to medical imaging.

On the UCSB dataset specifically, specialized computational approaches have achieved test accuracies ranging from approximately 90% with feature-engineering-based methods to above 96% with deep learning architectures [17,20,26]. The highest-performing MLLM in our study (Gemini 3.1 Pro, 99.3% under IMAGE) approached or matched these benchmarks, though the possible contribution of training data exposure complicates direct comparison. The remaining models performed substantially below these specialized baselines, consistent with the broader observation that general-purpose MLLMs underperform task-specific architectures on medical imaging tasks [8].

This study extends the modality-bias paradigm of Geirhos et al. [11] from CNNs to contemporary general-purpose MLLMs, applies a controlled within-case modality ablation separating visual and spatial information in breast histopathology, and jointly characterizes modality-dependent accuracy, confidence miscalibration, and input-dependent reasoning category shifts in this model class.

### 4.7. Limitations

Several limitations should be considered. First, the dataset comprised only 58 cases, which limits statistical power. However, the magnitude of the observed performance differences (up to 49 percentage points) is substantial relative to the sample size, and the five repeated runs per case provided within-case replication that increases measurement stability. Second, the segmentation masks represent partial annotations that do not capture all nuclei within each image and contain no information about tissue context. Even under these constrained structural representations, however, performance remained near-random, suggesting that the available spatial structural cues were not consistently leveraged. Third, as the UCSB dataset is publicly available, including through third-party mirrors on data-science platforms [28,29], prior exposure during model training cannot be excluded. This concern applies particularly to Gemini 3.1 Pro and is discussed in detail, together with evidence arguing against a pure memorization account, in Section 4.5. Fourth, ground truth labels were used as provided in the original benchmark dataset, consistent with their use across the extensive prior classification and segmentation literature on UCSB [13,14,15,16,17,18,19,20,21,22,23,24,25,26,27,28,29]. The within-case, within-model comparisons that constitute the core analysis are unaffected by label origin. Finally, all models were accessed via web interfaces under default settings, which does not allow for control of underlying sampling parameters such as temperature or top-p, and which may not reflect optimal performance achievable through API access, temperature tuning, or in-context learning strategies [5]. The findings are based on a single histopathology benchmark and a fixed set of model versions accessed in March 2026; generalization to other tissue types, imaging modalities, staining protocols, or future model versions remains to be empirically tested.

### 4.8. Implications and Future Directions

The convergence of modality dependence, limited spatial structural integration, confidence miscalibration, and input-dependent reasoning shifts suggests that current MLLMs do not perform visual–spatial integration in a manner analogous to human pathological assessment. Rather, classification behavior appears predominantly anchored in visual surface features, with spatial structural information neither independently utilized nor effectively combined with visual cues.

These findings have implications for the deployment of MLLMs in tasks where classification depends on spatial structural rather than visual surface cues, for instance in architectural pattern recognition, spatial distribution analysis, or contexts where staining variation reduces the reliability of texture features. In clinical pathology workflows, deployment of general-purpose MLLMs as primary diagnostic aids without specialized adaptation could lead to misdiagnosis in cases where structural rather than textural features are decisive, with the elevated confidence observed under MASK conditions illustrating the additional risk that such errors may not be flagged by the model itself. Several mitigation strategies merit systematic investigation: whether few-shot prompting with labeled examples can restore effective integration of spatial structural information under modality-constrained inputs; whether fine-tuning on modality-manipulated training pairs can reduce modality dependence; whether the observed behavioral patterns generalize beyond breast histopathology when the feature-ablation framework is applied to other imaging modalities and tissue types; and whether direct comparison against specialized CNN architectures under identical evaluation protocols closes or preserves the performance gap observed against published benchmarks. Hybrid architectures that combine the broad reasoning capabilities of general-purpose MLLMs with domain-specific visual encoders represent a particularly promising direction, potentially preserving the flexibility of natural-language interfaces while compensating for the modality-integration limitations identified here [34].

## 5. Conclusions

This study provides evidence that six contemporary general-purpose MLLMs, under the conditions tested, exhibit strong dependence on the visual information present in H&E-stained images for breast cancer histopathological classification. Mean accuracy collapsed from 69.4% on H&E images to 49.6%, below the majority-class baseline of 55.2%, when only spatial information from nuclei segmentation masks was provided. Adding the mask to the image did not improve classification and, for some models, actively degraded performance, despite the mask containing additional information that could only have been supplementary to the visual content. Confidence remained elevated under near-random performance, with no meaningful discrimination between correct and incorrect predictions, and reasoning categories shifted in 67.5% of matched case–run pairs between modalities without any change to the underlying diagnostic question. Taken together, these four observations converge on a single pattern: model behavior was driven by the format and content of the input representation rather than by a stable, modality-independent interpretation of the underlying pathology. The observed modality dependence extends the texture-bias paradigm previously established for convolutional neural networks to contemporary MLLMs, and together with published performance of specialized approaches exceeding 96% accuracy on the same dataset underscores the continued relevance of domain-specific model designs for cancer histopathology. Rigorous behavioral characterization of modality dependence, confidence calibration, and reasoning stability should routinely precede deployment of general-purpose MLLMs in any diagnostic context that requires reliable visual–spatial integration.

**What are the main findings?**

- Six contemporary general-purpose multimodal large language models (MLLMs) from Anthropic, OpenAI, xAI, and Google showed strong modality dependence in breast cancer histopathology, with mean classification accuracy collapsing from 69.4% on hematoxylin and eosin (H&E)-stained images to 49.6%, below the majority-class baseline, when only nuclei segmentation masks were provided.
- When H&E images and segmentation masks were provided together, classification did not improve over images alone and in some cases worsened. This indicates that current MLLMs fail to effectively integrate visual and spatial structural information for cancer classification.

**What are the implications of the main findings?**

- Models maintained elevated confidence under near-random performance regimes and shifted their self-reported reasoning categories depending on input modality, revealing systematic miscalibration and reasoning instability with direct implications for the safe deployment of general-purpose MLLMs in cancer diagnostic support.
- Contemporary general-purpose MLLMs do not yet achieve the level of visual–spatial integration required for reliable breast cancer histopathological classification, while specialized deep learning approaches reported in the literature exceed 96% accuracy on the same dataset, highlighting the continued need for domain-specific model designs in AI-driven cancer research.

## Figures and Tables

**Table 1:** Experimental conditions and input specifications.

Condition	Input	Available Information
IMAGE	H&E-stained histopathology image	Full visual information: tissue architecture, nuclear morphology, cell distribution, staining patterns
MASK	Binary segmentation mask only	Spatial distribution of annotated regions; no color, texture, or nuclear detail
BOTH	H&E image + segmentation mask	Full visual information plus explicit spatial annotation overlay

The dataset comprised 58 H&E-stained breast histopathology cases (26 malignant, 32 benign; majority-class baseline 55.2%). Under each condition, models produced a forced output consisting of a binary label (malignant or benign), a confidence score (0–100), and a reasoning category selected from five predefined options (Nuclear features, Global structure, Texture, Cell distribution, or Uncertain guess).

**Table 2:** Classification accuracy by model and condition.

Model	IMAGE % (95% CI)	MASK % (95% CI)	BOTH % (95% CI)	*p* ^1^	*p* ^2^
Claude Opus 4.6	67.6 (62.0–72.7)	46.9 (41.2–52.6)	63.8 (58.1–69.1)	0.0081	0.6072
Claude Sonnet 4.6	63.8 (58.1–69.1)	52.4 (46.7–58.1)	64.1 (58.5–69.4)	1.0000	1.0000
Claude Haiku 4.5	54.8 (49.1–60.5)	50.3 (44.6–56.1)	59.3 (53.6–64.8)	0.8450	0.4807
ChatGPT 5.3	83.8 (79.1–87.6)	49.7 (43.9–55.4)	73.1 (67.7–77.9)	0.0003 *	0.4531
Grok 4.2 †	47.2 (41.6–53.0)	47.9 (42.2–53.7)	47.6 (41.9–53.3)	0.6250	1.0000
Gemini 3.1 Pro	99.3 (97.5–99.8)	50.3 (44.6–56.1)	100.0 (98.7–100.0)	<0.0001 *	1.0000

Accuracy and 95% Wilson confidence intervals computed at the run level (*n* = 290 per cell). *p*^1^ = IMAGE vs. MASK; *p*^2^ = IMAGE vs. BOTH (McNemar’s exact test on majority-vote, *n* = 58). * Significant after Bonferroni correction (α = 0.0042). † Near-constant malignant classifier.

**Table 3:** Diagnostic performance (%) by model and condition.

Model	Cond.	Sens %	Spec %	PPV %	NPV %	Bal. Acc %
Claude Opus 4.6	IMAGE	84.6	62.5	64.7	83.3	73.6
Claude Opus 4.6	MASK	69.2	28.1	43.9	52.9	48.7
Claude Opus 4.6	BOTH	88.5	50.0	59.0	84.2	69.2
Claude Sonnet 4.6	IMAGE	100.0	34.4	55.3	100.0	67.2
Claude Sonnet 4.6	MASK	80.8	46.9	55.3	75.0	63.8
Claude Sonnet 4.6	BOTH	96.2	37.5	55.6	92.3	66.8
Claude Haiku 4.5	IMAGE	26.9	78.1	50.0	56.8	52.5
Claude Haiku 4.5	MASK	50.0	53.1	46.4	56.7	51.6
Claude Haiku 4.5	BOTH	26.9	90.6	70.0	60.4	58.8
ChatGPT 5.3	IMAGE	100.0	68.8	72.2	100.0	84.4
ChatGPT 5.3	MASK	46.2	50.0	42.9	53.3	48.1
ChatGPT 5.3	BOTH	100.0	59.4	66.7	100.0	79.7
Grok 4.2 †	IMAGE	100.0	0.0	44.8	—	50.0
Grok 4.2 †	MASK	96.2	9.4	46.3	75.0	52.8
Grok 4.2 †	BOTH	100.0	0.0	44.8	—	50.0
Gemini 3.1 Pro	IMAGE	100.0	100.0	100.0	100.0	100.0
Gemini 3.1 Pro	MASK	50.0	50.0	44.8	55.2	50.0
Gemini 3.1 Pro	BOTH	100.0	100.0	100.0	100.0	100.0

All metrics computed on majority-vote predictions (*n* = 58). † Near-constant malignant classifier; NPV undefined (no true negative predictions) for Grok 4.2 under IMAGE and BOTH conditions.

**Table 4:** Pairwise label change analysis, BOTH versus IMAGE condition.

Model	Changed (%)	Improved	Worsened	Net
Claude Opus 4.6	99 (34.1%)	44	55	−11
Claude Sonnet 4.6	87 (30.0%)	44	43	+1
Claude Haiku 4.5	107 (36.9%)	60	47	+13
ChatGPT 5.3	53 (18.3%)	11	42	−31
Grok 4.2 †	47 (16.2%)	24	23	+1
Gemini 3.1 Pro	2 (0.7%)	2	0	+2

Each model contributed 290 matched case–run pairs. Improved = incorrect in IMAGE, correct in BOTH; Worsened = correct in IMAGE, incorrect in BOTH; Net = Improved − Worsened. † Near-constant malignant classifier.

## Data Availability

All data analyzed in this study are publicly available and described in the Introduction and Materials and Methods Sections [18,28,29].

## Supplementary Materials

The following supporting information can be downloaded at: https://www.mdpi.com/article/10.3390/life16050763/s1, Supplementary Material S1: Full Inference Prompt.

## Abbreviations

The following abbreviations are used in this manuscript:

AI	Artificial Intelligence
API	Application Programming Interface
AUC	Area under the curve
CI	Confidence Interval
CNN	Convolutional Neural Network
H&E	Hematoxylin and Eosin
LLMs	Large Language Models
MLLM	Multimodal Large Language Model
NPV	Negative Predictive Value
PPV	Positive Predictive Value
SD	Standard Deviation
