# Supplementary material for: Artificial Intelligence in Cancer Research: Modality Dependence and Limited Visual–Spatial Integration in Multimodal Large Language Models for Breast Cancer Histopathology
Source: Life (Basel). 2026 May 2;16(5):763. doi: 10.3390/life16050763 (PMC13208433; doi:10.3390/life16050763)
Supplement: Supplementary file 1 [file life-16-00763-s001.zip › Supplementary Material S1 Full Inference Prompt.pdf]

Supplementary Material S1 Full Inference Prompt

You are presented with a histopathological hematoxylin and eosin (H&E)-stained image of breast tissue, a corresponding nuclei segmentation mask, or both. Your task is to classify the case as either malignant or benign based solely on the provided input. Follow the instructions strictly.

Provide your output in the exact format below, with each component on its own line and no additional text:

Classification: <malignant | benign>

Confidence: <integer 0–100>

Reasoning: <Nuclear features | Global structure | Texture | Cell distribution | Uncertain guess>

Provide exactly one classification, exactly one integer confidence value between 0 and 100, and select exactly one reasoning category. Do not include any additional explanation or commentary.
